# Assessment of Gaps and Inequalities in Cancer Screening at the District Level in Peru

**DOI:** 10.3390/epidemiologia6040074

**Published:** 2025-11-04

**Authors:** Akram Hernández-Vásquez, Lucía Villar Bernaola, Maricela Curisinche-Rojas, Raúl Timaná-Ruiz

**Affiliations:** Subdirección de Investigación de Servicios de Salud (SDISS), Centro de Evaluación de Tecnologías en Salud (CETS), Instituto Nacional de Salud (INS), Lima 15066, Peru; lvillar@ins.gob.pe (L.V.B.); mcurisinche@ins.gob.pe (M.C.-R.); rtimana@ins.gob.pe (R.T.-R.)

**Keywords:** neoplasms, cancer screening test, health status disparities, Peru

## Abstract

**Objectives**: This study assessed socioeconomic inequalities in cancer screening at the district level in Peru, focusing on bilateral mammography, fecal occult blood test (FOBT), and prostate-specific antigen (PSA) test. **Methods**: An ecological study was conducted using 2021–2023 data from the Health Information System (HIS) of MINSA. Screening rates were calculated per 1000 eligible individuals. Socioeconomic disparities were assessed using concentration indices (CIs) and gap analysis, with the Human Development Index (HDI) as the stratification variable. **Results**: Screening rates were higher in districts with greater HDI. The mean district-level rates were 15.41 (SD: 72.66) for mammography, 97.27 (SD: 107.34) for FOBT, and 104.87 (SD: 101.92) for PSA per 1000 eligible individuals. Positive concentration indices indicated a pro-rich inequality: CI for mammography (0.1745, *p* = 0.045), FOBT (0.0633, *p* < 0.001), and PSA (0.0290, *p* = 0.028). The largest gaps were observed in Amazonian and Andean regions, where screening coverage remained markedly low. Spatial distribution revealed that certain districts, particularly in Loreto, Ucayali, and Amazonas, had screening gaps exceeding 97%. **Conclusions**: Significant disparities in cancer screening exist across Peruvian districts, disproportionately affecting lower-HDI districts areas. Targeted interventions, including education, telemedicine, and improved infrastructure, are necessary to enhance equitable access to early detection services and reduce the burden of disease.

## 1. Introduction

Cancer is a non-communicable disease that poses a substantial public health challenge, as it remains one of the leading contributors to the global burden of disease [1]. In 2019, cancer was responsible for 10 million deaths and 250 million disability-adjusted life years (DALYs), reflecting a 20.9% and 16.0% increase, respectively, compared to 2010 [1]. Although cancer incidence is higher in high-income countries compared to low- and middle-income countries (LMICs), cancer-related mortality is greater in LMICs [2,3]. In Latin America and the Caribbean (LAC), over one million people were newly diagnosed with cancer in 2020, accounting for 7.6% of all new cancer cases worldwide, and approximately 700,000 people died from the disease that year [4]. In 2022, Peru ranked eleventh in LAC for cancer incidence rates (174 new cases per 100,000 inhabitants) and tenth for cancer mortality rates (with 82 deaths per 100,000 inhabitants) [5]. These alarming figures underscore a major challenge for public health efforts in cancer control, driven by the high costs and delays in diagnosis and treatment, especially in regions with limited access to healthcare services.

Although cancer has high incidence and mortality rates, nearly half of all cancer types are preventable [6], and 70–80% of cases potentially preventable are concentrated in low- and middle-income countries (LMICs) [2]. Preventive measures for cancer control focus on reducing risk factors (through changes in diet, physical activity, and harmful habits), timely and appropriate treatment, and early screening [7]. However, in LMICs, screening programs face individual- and system-level barriers, such as low coverage, insufficient human resources, and a lack of necessary supplies for diagnostic tests, along with delays in referring patients to oncology centers [8]. Similar structural barriers are observed in Peru, as in other LMICs [8]. The main barriers include unequal healthcare personnel and infrastructure distribution, extended travel and wait times, and insufficient access to health insurance [9]. On an individual level, sociodemographic factors such as income and education are positively associated with participation in cancer screening programs in LMICs [10]. Furthermore, lack of health insurance and residing in rural areas are factors that further hindered participation in these programs.

Cancer screening programs in Peru focus primarily on cervical and breast cancer, implemented nationwide but with uneven coverage and access [11]. The cervical cancer screening program relies predominantly on Papanicolaou testing under MINSA, with VIA (visual inspection with acetic acid) used in remote settings [12]. Breast cancer screening is limited to mammography for women aged 40–69 every two to three years and clinical breast examination [12], but implementation has been uneven. In many rural areas or departments, the mammography infrastructure is sparse, services are mostly opportunistic (only screening patients who visited the practice), and CBE is used when mammography is not accessible [13,14,15]. National data from recent Demographic and Family Health Surveys (ENDES) suggest that cervical cancer screening (Papanicolaou testing) coverage among women in target ages has reached around 70% in many regions [16], whereas mammography coverage remains substantially lower, especially in rural or remote regions [16]. Although these programs are formally organized and population-based under national policy frameworks, in practice screening largely occurs when individuals interact with the health system for other reasons or upon request. Population-based efforts remain partial and heterogeneous, with significant variation across regions depending on resources, infrastructure, and service capacity.

In Peru, several population studies, based on ENDES data, have explored the socioeconomic inequalities in cancer screening tests, reporting that people with higher socioeconomic status have a greater prevalence of screening for early detection of breast and cervical cancer [17,18]. This evidence underscores the disparities that the Peruvian population faces in accessing cancer screening tests, which hinder progress toward achieving universal health coverage as outlined in the Sustainable Development Goals (SDG) 3.8 and 3.4 (reducing premature mortality from non-communicable diseases by one-third by 2030) [19]. However, although ENDES collects annually information for women on clinical breast examination, Pap smear test, and mammography [16], it does not collect data on other screening tests for women and men such as the fecal occult blood test, nor for men such as the prostate-specific antigen test. Furthermore, there is a current deficit and uneven geographic distribution of mammography machines in Peru. For example, in 2017, only 37 out of 52 reported mammography machines nationwide were operational [20]. By 2025, the number of operational mammographs in the public health system has increased to 61 [21], although many departments still have no mammograph at all, with most units concentrated in large cities.

To address this gap research, we aimed to determine the gaps and inequalities in cancer screening at the district level in Peru, focusing on the population served by public health services under the Regional Governments and the Ministry of Health. In Peru, according to recent estimates from GLOBOCAN 2022, the cancers with the highest incidence and mortality rates are prostate, breast, stomach, colorectal, and cervical cancer [22]. Therefore, our study focuses on the period 2021–2023 and examines three types of screening available through the public system: bilateral screening mammography, fecal occult blood test, and prostate-specific antigen, which address the cancers with the highest burden in the Peruvian population. We selected this period to characterize the post-pandemic status of screening services and to assess current inequalities.

## 2. Materials and Methods

### 2.1. Data Source

An ecological study was conducted using administrative data from the Health Information System (HIS, Spanish acronym) databases of the Ministry of Health of Peru (MINSA). This system includes records of healthcare services provided in public health services, covering approximately 63.5% of the Peruvian population [23]. These databases, covering the years 2021, 2022, and 2023, were requested from the General Office of Information Technologies at the Ministry of Health under the provisions of the Transparency and Access to Public Information Law.

The datasets were explicitly provided for each CPT code and segmented according to the target population’s sex and age. Data were requested for the following prioritized screening services and their respective population groups [12]: (1) Bilateral Screening Mammography (CPT 77057): Women aged 40 to 69; (2) Fecal Occult Blood Test (FOBT, CPT 82270): people aged 50 to 70; and (3) Prostate-Specific Antigen (PSA) Test (CPT 84152): Men aged 40 to 75.

Each database contained detailed information on anonymized unique identifiers for each patient attended, including appointment details, individual and healthcare facility identifiers, year, month, department name, province name, district name, geographic location code, classification according to the National Registry of Accredited Health Establishments (RENAES, Spanish acronym), the Integrated Health Networks Directorate (DIRIS, Spanish acronym) to which the health facility belongs, health facility description, Current Procedural Terminology (CPT) code and description, sex, and age.

### 2.2. Inclusion and Exclusion Criteria

This study included records of individuals who underwent at least one screening test, based on their place of residence. Individuals were excluded if their record was listed only as a referral during the study period, as well as duplicate records, where two appointments were scheduled on the same day at the same facility and for the same service.

### 2.3. Variables and Measures

The rates of the three prioritized screening tests were calculated per 1000 persons for the study period (2021–2023), which represents the post-pandemic phase, when health services began to recover their operational capacity after the COVID-19 restrictions. While the screening test rates are typically calculated on an annual basis, the three-year period was considered as it reflects a post-pandemic phase during which health services had not yet returned to full operational capacity following the COVID-19 lockdown. Regarding the numerator, the number of individuals was identified by district of residence who had undergone at least one of the three prioritized screening services. The numerator was constructed from the HIS database, considering unique and anonymized patient identifiers. Meanwhile, the denominator was calculated from the number of active Seguro Integral de Salud (SIS) members as of June 2022, which was downloaded from the National Open Data Platform of the Peruvian government [24]. The SIS covers approximately 65% of the Peruvian population [23]. The selection of June 2022 aimed to provide a point estimate of the SIS population at a midpoint during the study period.

In addition, the Human Development Index (HDI) was obtained from data published by CEPLAN (Centro Nacional de Planeamiento Estratégico) [25]. The HDI is constructed using three key indicators: life expectancy at birth, the proportion of the population over 18 with secondary education, and per capita household income. HDI values close to 1 indicate higher levels of human development within a given territory. Data from 2019 were used to determine the district-level HDI, which was considered a socioeconomic stratification variable for each district included in the study.

### 2.4. Statistical Analysis

All analyses were performed using StataNow/SE 18.5 (StataCorp, College Station, TX, USA). A two-tailed *p*-value < 0.05 was considered statistically significant.

Numerical data, including screening test rates and the HDI, were summarized using mean and standard deviation (SD), as well as median and interquartile range (IQR) for the total number of districts included in the analysis. In addition, inequality and gap analyses were conducted. Inequalities in screening tests were assessed using concentration curves (CC) and indices (CI), with the HDI serving as the economic stratification variable. CCs describe the cumulative percentage of screening tests in relation to the cumulative percentage of the population, ordered by HDI from lowest to highest. The equality line in the CC is a diagonal drawn from the bottom left corner to the top right corner, representing perfect equality. If the outcome is higher (or lower) among the lowest-developed districts, the CC will be above (or below) the equity line. Conversely, if all districts have the same outcome value regardless of HDI, the CC will coincide with the equity line. The further the CC is above the equity line, the greater the concentration of the outcome among the lowest-developed districts, and the further it is below, the greater the concentration among the highest-developed districts. Moreover, CI is a relative measure of inequality defined as twice the area between the concentration curve (CC) and the line of equity. For this analysis, the generalized concentration index was considered, given that the outcome variable is numerical (screening test rate). The index ranges from μ–1 to 1–μ. If the CC lies above (or below) the equity line, the concentration index will take on a positive (or negative) value. The greater the absolute value of the index, the higher the level of socioeconomic inequality.

Furthermore, gap analysis was conducted using the absolute Kuznets index. This index was calculated by subtracting the screening test rate of districts in the lowest quintile of the HDI from the screening test rate of districts in the highest HDI quintile. Additionally, the district gap was calculated to determine the proportion of the target population that remains unscreened to achieve 100% screening coverage. The following equation was used for this calculation:

x = (1 − (A/B)) × 100

where A represents the number of individuals who underwent at least one screening and were registered in the HIS, while B represents the number of individuals in the target population who were affiliated with the SIS in the district. The result was multiplied by 100 to express the estimate as a percentage. These percentages were spatially represented by grouping the gaps into quintiles based on district indicators, as well as identifying districts where no screening was recorded. For the latter, the geographic information system software QGIS was used.

### 2.5. Ethical Aspects

Ethics committee approval was not required for this study, as it involved the analysis of aggregated secondary data from publicly available sources, which do not permit the identification of individual participants.

## 3. Results

We included district-level data from three screening tests in the analysis. For bilateral screening mammography, 977 districts were included. The mean district-level rate for this test was 15.41 tests (SD: 72.66) per 1000 women aged 40–69 years, with an average district HDI of 0.45 (SD: 0.15). For the FOBT and PSA testing, 1820 and 1814 districts, respectively, were included. The former had a mean district-level screening rate of 97.27 (SD: 107.34) per 1000 people aged 50–70 years, while the latter showed a mean rate of 104.87 (SD: 101.92) per 1000 men aged 40–75 years. Both tests had an average district HDI of 0.41 (SD: 0.14). Further details can be found in Table 1.

**Figure 1:**
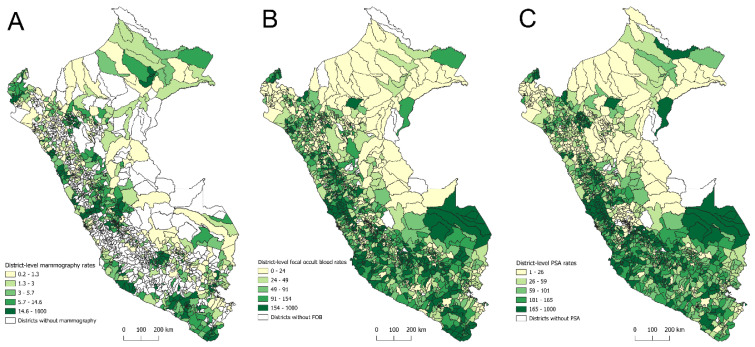
Spatial distribution of district-level screening rates for bilateral mammography (**A**), fecal occult blood test (**B**), and prostate-specific antigen test (**C**) in Peru, 2021–2023.

Figure 1A–C show the spatial distribution of district-level rates for the three screening tests. Overall, the highest rates of bilateral screening mammography were observed in districts within coastal departments such as Lima, Tacna, Moquegua, Ica, and Arequipa, ranging from 14.6 to 1000 tests per 1000 women aged 40–69 years. Conversely, the lowest rates (i.e., 0.2 to 1.3 tests per 1000 women aged 40–69 years) were found in districts within departments such as Lambayeque, Piura, Cajamarca, and Puno (Figure 1A). For the FOBT, the highest rates (i.e., 154 to 1000 tests per 1000 people aged 50–70 years) were observed in districts within coastal departments such as Tumbes, Lambayeque, La Libertad, Áncash, Lima, Ica, and Arequipa, as well as in Amazonian departments such as Madre de Dios and San Martín. In contrast, the lowest rates (i.e., 0 to 24 tests per 1000 people aged 50–70 years) were found in districts of Amazonian departments such as Loreto and Ucayali (Figure 1B). Regarding the PSA test, the highest rates (i.e., 165 to 1000 tests per 1000 men aged 40–75 years) were recorded in districts within Madre de Dios and coastal departments such as Lima, Ica, Arequipa, and Moquegua. Districts with the lowest PSA rates (i.e., 1 to 26 tests per 1000 men aged 40–75 years) were located in Amazonian departments such as Amazonas, Loreto, San Martín, and Ucayali, as well as in the Andean department of Junín and the coastal department of Piura (Figure 1C).

**Figure 2:**
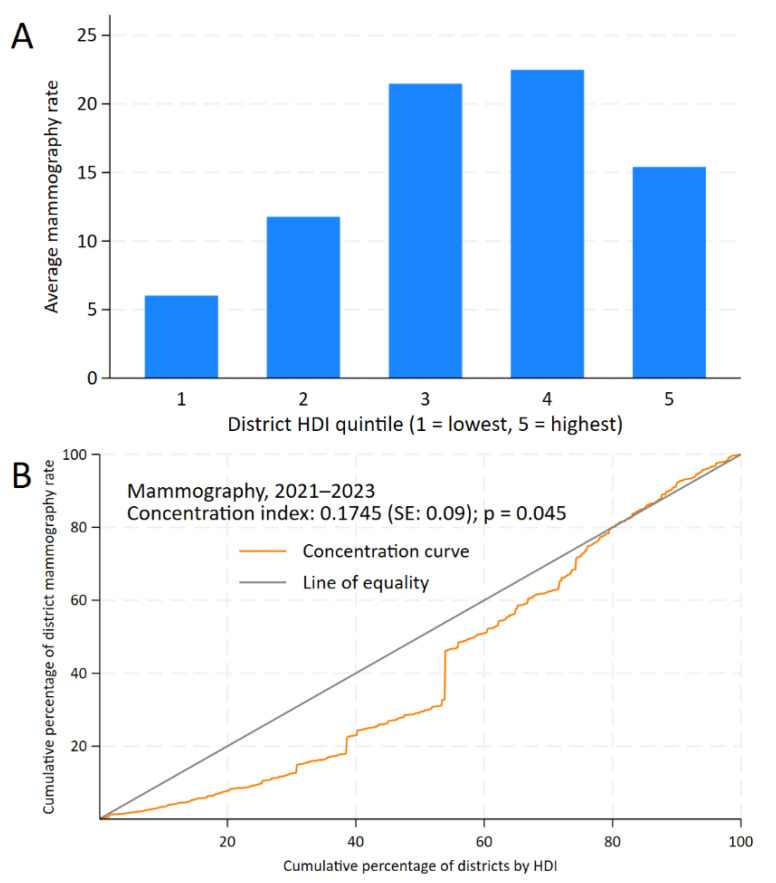
Inequality analysis of screening test uptake by district-level Human Development Index (HDI) in Peru, 2021–2023. Kuznets index for bilateral mammography (**A**) and concentration curves and indices for bilateral mammography (**B**).

In the inequality analysis, the CCs indicate that bilateral screening mammography, FOBT, and PSA testing were concentrated in districts with higher HDI levels. For bilateral screening mammography, the CC remains below the line of equality between 0% and 80% of the cumulative percentage of districts by HDI, showing lower cumulative rates in districts with lower HDI than those with higher HDI. Beyond this point, the distribution approaches equality. This is reflected in the positive CI for bilateral screening mammography (0.1745, standard error: 0.09; *p* = 0.045) and the gap between districts of +9.39 (95% CI: 5.64 to 13.14; *p* < 0.001), as shown in Figure 2.

**Figure 3:**
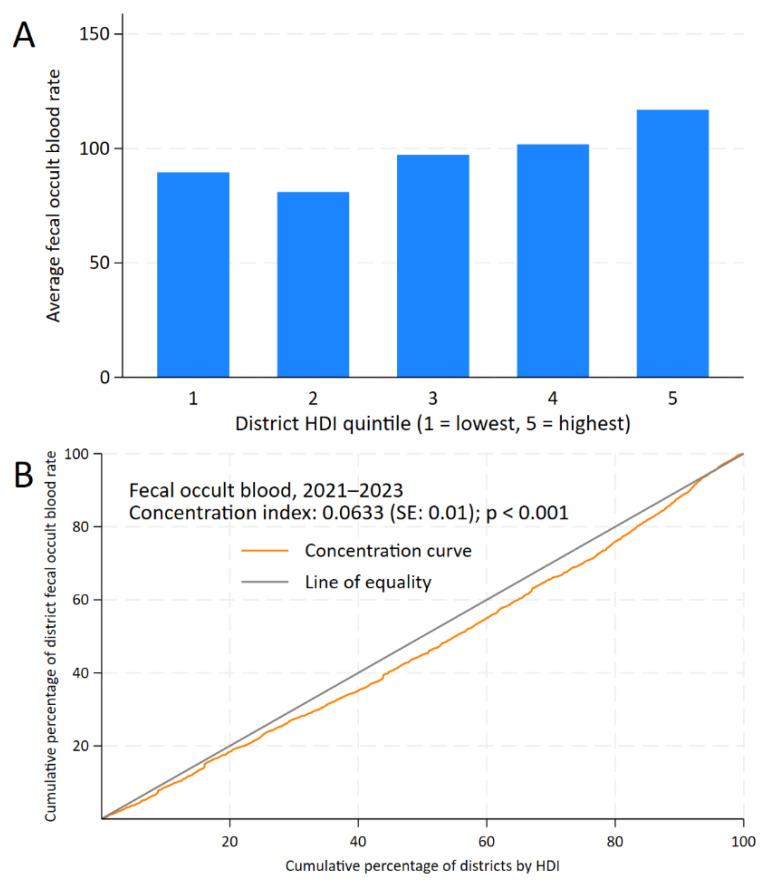
Inequality analysis of screening test uptake by district-level Human Development Index (HDI) in Peru, 2021–2023. Kuznets index for fecal occult blood test (**A**) and concentration curves and indices for fecal occult blood test (**B**).

For FOBT, the CC remains below the line of equality up to 90% of the cumulative percentage of HDI, with a positive CI of 0.0633 (standard error: 0.01; *p* < 0.001) and a district gap of +27.26 (95% CI: 10.54 to 43.99; *p* = 0.001) (Figure 3).

**Figure 4:**
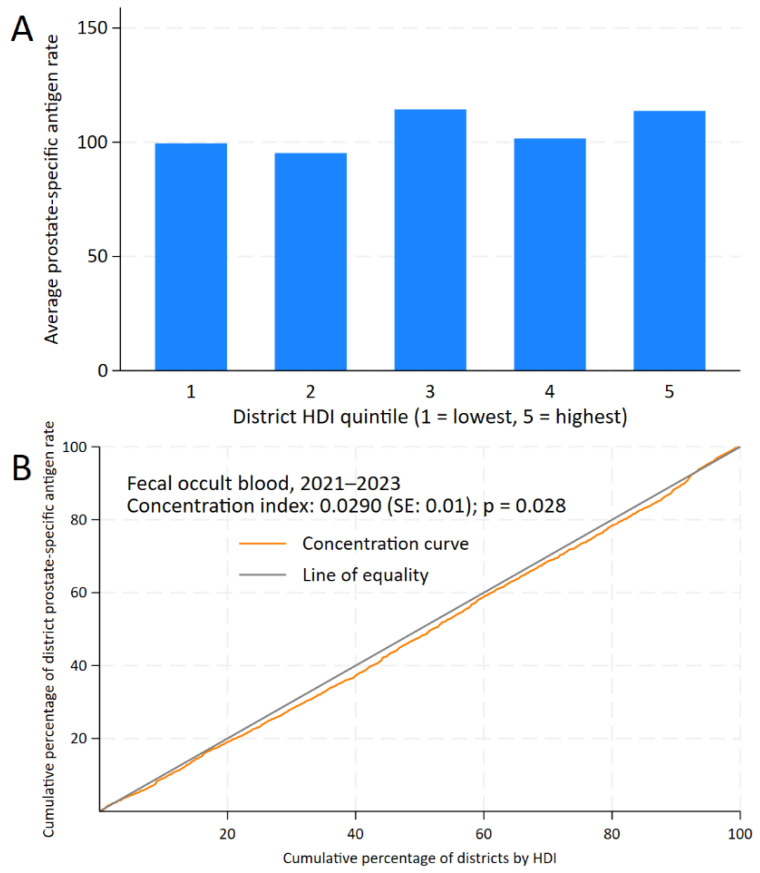
Inequality analysis of screening test uptake by district-level Human Development Index (HDI) in Peru, 2021–2023. Kuznets index for prostate-specific antigen test (**A**) and concentration curves and indices for prostate-specific antigen test (**B**).

For PSA testing, while inequality is less pronounced, the CC still lies below the line of equality. The positive CI of 0.0290 (standard error: 0.01; *p* = 0.028) indicates a concentration of PSA testing in districts with higher HDI, and the gap between districts was +14.19 (95% CI: −1.20 to 29.57; *p* = 0.071), as shown in Figure 4.

**Figure 5:**
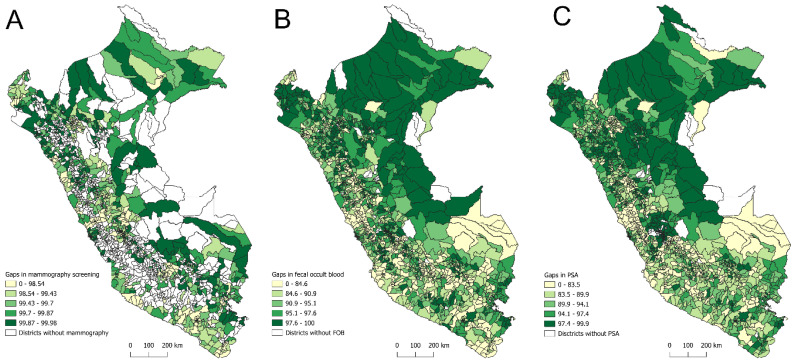
Spatial distribution of district-level screening gaps for bilateral mammography (**A**), fecal occult blood test (**B**), and prostate-specific antigen test (**C**) in Peru, 2021–2023.

Regarding the spatial distribution of district-level gaps for the three screening tests, the largest gaps in bilateral screening mammography (99.87% to 99.98%) were concentrated in districts from Highlands such as Huánuco, Puno, and Cusco; Amazonian departments, including Loreto, Amazonas, San Martín, and Ucayali; and coastal departments such as Piura and Lambayeque (Figure 5A). For FOBT, the most significant gaps (97.6% to 100%) were also observed in Amazonian departments (Loreto, Amazonas, San Martín, and Ucayali), along with the Andean department of Cajamarca and the coastal department of Piura (Figure 5B). Similarly, PSA testing exhibited pronounced gaps (97.4% to 99.9%) in Amazonian departments (Loreto, Amazonas, and San Martín), Highlands (Huánuco and Junín), and the coastal department of Piura (Figure 5C).

## 4. Discussion

Bilateral screening mammography, FOBT, and PSA test rates showed higher concentrations in districts with higher HDI than those with lower HDI. This pattern was also reflected in the positive values of the Kuznets Index gaps between districts with higher and lower HDI levels. These results support the importance of achieving Sustainable Development Goal 3.4, which aims to reduce mortality from non-communicable diseases through preventive strategies [26] and the World Health Organization’s Global Breast Cancer Initiative goals [27].

Population-screening tests such as bilateral screening mammography, FOBT, and PSA are highly recommended for the detection of pre-cancerous changes or early-stage breast, colorectal, and prostate cancers [28,29,30,31]. Early detection is particularly important in Peru, as these three types of cancer are among the top seven leading causes of cancer-related mortality [5]. However, inequalities in access to screening tests may lead to delayed diagnosis and treatment for high-risk populations, significantly impacting survival rates and long-term clinical outcomes [32]. Given that these tests are, on average, conducted more frequently in districts with higher HDI compared to those with lower HDI, the burden of cancer is likely to impact the latter disproportionately. In the context of advanced-stage cancer diagnoses arising from unequal access to screening tests, the importance of early detection through equitable screening programs becomes increasingly apparent, offering both economic and clinical benefits by reducing treatment costs and improving patient outcomes [33,34].

In Peru, these inequalities have been documented in epidemiological studies, although primarily at the individual level. For example, Intimayta-Escalante provided evidence that mammography screening is more concentrated among women with higher wealth indices than those with lower indices [16]. Similarly, Hernández-Vásquez et al. found that as the wealth index increased, the prevalence ratio of mammography screening also rose [35]. Notably, this pro-rich pattern is not unique to Peru and has also been reported in other low- and middle-income countries [10,36]. While economic inequalities are a key factor driving these disparities, other system- and individual-level barriers (e.g., education, knowledge, area of residence, among others) also play a critical role [16,18,35]. For PSA, a previous observational study on prostate cancer screening in low- and middle-income countries showed that the prevalence of prostate cancer screening was higher among people with a higher wealth index compared to those with a lower wealth index in countries such as Namibia, Honduras, and the Dominican Republic [37]. Although the results of these studies may be subject to misclassification bias due to self-reporting, our analysis, based on medical reports, shows similar findings, highlighting the consistency of results at the district level. For FOBT, a previous systematic review on colorectal cancer screening identified a similar trend, consistent with our study [38], showing that people in higher or middle-income areas are more likely to participate in screening than those in the poorest areas.

Another key result of this study is the district-level distribution of bilateral screening mammography, FOBT, and PSA testing rates. Although the spatial analysis did not identify a consistent district-level pattern across the screening tests, a greater concentration of districts with lower screening rates was observed in the Coastal and Amazon regions. For bilateral screening mammography, the lowest rates were found in districts within departments such as Lambayeque, Piura, Cajamarca, and Puno. For FOBT and PSA, the lowest rates were observed in Loreto and Ucayali, and Amazonas, Loreto, San Martín, and Ucayali, respectively. These results are similar to those reported in 2022 and 2023 by the Observatory for Cancer Prevention and Control Interventions of the Ministry of Health [39]. The lower screening rates in the Amazon region contributed to larger district-level coverage gaps within the same area. Various geospatial factors influence the gaps in cancer screening tests in this region. These factors are related to the reach of community health workers, educational programs, communication about screening campaigns, and the distance of homes from health centers offering the tests [40]. The latter is also a factor observed in rural areas of other contexts, where distance and lack of transportation pose a barrier to the success of screening test programs [41,42]. Given that the Amazon region is home to the largest number of native populations [43], another relevant factor is individuals’ knowledge and beliefs regarding these tests, as shown in other contexts [44]. The lack of a culturally sensitive approach to education about the tests and distrust in the healthcare system are potential contributors to the barriers associated with knowledge and beliefs [44]. In addition to geographical and cultural barriers in the Amazon, in the Andean region, altitude and difficult road access constrain travel to health facilities and medical consultations in Peru [45,46]. Added to this are sociocultural barriers related to healthcare among Quechua- and Aymara-speaking communities living in the Andean region of Peru [47,48]. To move forward, qualitative studies are needed to investigate local perceptions, and a review of regional policies is required to allocate resources equitably according to the sociocultural characteristics of the population.

Another key aspect to emphasize is the calculation of the indicator. In several districts, the screening rate did not exceed one, highlighting that many districts had very few individuals undergoing screening tests. Specifically, regarding bilateral mammography rates, it is essential to note that Peru has limited mammography equipment predominantly located in referral hospitals or capital cities [14]. Additionally, some of these facilities are inoperative, further restricting access to this screening service [14]. This reflects how the shortage of economic and human resources may significantly impede the implementation and promotion of secondary prevention screening tests. Furthermore, the successful completion of screening tests depends on the availability of preventive tools and individuals’ willingness and ability to participate in these tests [49,50]. In this regard, addressing the gaps in access to cancer screening nationwide, increasing the availability of tests, encouraging public participation in prevention strategies, and enhancing knowledge and beliefs among people will allow for the reformulation of existing strategies by the health system, improving structural conditions and access to measures that prevent the rising burden of cancer.

This analysis has limitations. First, the study was based on a single data source containing information on people treated in healthcare centers and establishments under the Ministry of Health (MINSA). This limits the generalizability of the data to the broader population, as the health system comprises various subsystems, such as EsSalud, Armed Forces, Police, and Private Insurance, which cover approximately 35% of the Peruvian population [23]. Second, the data originate from the HIS (a specific format filled out during medical consultations) and rely on the documentation provided by the treating physician. This documentation may contain transcription errors or inappropriate coding of the procedures performed, resulting in inadequate or unreliable data. In addition, HIS is a healthcare data collection system whose completeness and quality may vary depending on the operational capabilities of healthcare facilities, and may be of lower quality in areas with lower HDI. Third, the indicators for each of the screening tests at the district level may have a numerator with a limited number of observations, potentially underestimating the true estimate. These limitations in the numerator may bias the results (either underestimating or overestimating) and present an unreliable picture. Fourth, the denominator was calculated based on the central year (2022) of the included time horizon (2021–2023). This calculation does not account for changes in an individual’s SIS affiliation status over the year. Such changes in affiliation could affect the results presented (leading to underestimation or overestimation). Fifth, some district-level rates could not be obtained for all Peruvian districts due to a lack of data on indicators, particularly regarding recorded consultations in the HIS. Sixth, although the HDI was the main stratifier, we recognize that it does not cover factors such as multidimensional poverty, geographic accessibility, and cultural beliefs. Future studies should use a social determinants of health approach, integrating individual and district variables, and considering composite indices that reflect the complexity of inequalities in cancer screening. Finally, our study, based on an ecological design of aggregated data at the district level, is subject to ecological fallacy, which prevents inferences from being made at the individual level. Despite these limitations, determining these indicators based on MINSA data (covering 65% of the population) will allow for an appropriate restructuring of health prevention systems and help reduce the access gap to these services.

## 5. Conclusions

In conclusion, the district’s HDI shows significant inequalities in access to screening tests. Moreover, several districts, particularly those in the Amazon region, face the largest gaps in the uptake of bilateral screening mammography, FOBT, and PSA. Given that these tests are recommended for screening cancers with a high disease burden, screening programs must prioritize improving access through targeted and effective strategies such as culturally sensitive educational campaigns, telemedicine, and subsidies or financial incentives. Moreover, strengthening healthcare infrastructure and expanding the workforce of trained professionals are essential steps toward reducing these disparities and ensuring universal screening access. Ultimately, closing these gaps is not only an ethical imperative but also a cost-effective strategy to reduce cancer-related morbidity and mortality. Success will require political commitment, prioritized funding, and intersectoral collaboration to address the social determinants of health at the policy level.

## Figures and Tables

**Table 1:** Descriptive characteristics of the three indicators included in this study.

**Item**	**Value**
Bilateral screening mammography	
Number of districts with screened women	977
District-level rate	
Mean, SD	15.41 (72.66)
Median, Q25 and Q75	4.03 (1.59 and 10.91)
Human Development Index	
Mean, SD	0.45 (0.15)
Median, Q25 and Q75	0.45 (0.34 and 0.57)
Fecal occult blood	
Number of districts with screened people	1820
District-level rate	
Mean, SD	97.27 (107.34)
Median, Q25 and Q75	67.69 (29.20 and 136.55)
Human Development Index	
Mean, SD	0.41 (0.14)
Median, Q25 and Q75	0.39 (0.31 and 0.51)
Prostate-specific antigen dosage	
Number of districts with screened men	1814
District-level rate	
Mean, SD	104.87 (101.92)
Median, Q25 and Q75	78.60 (34.58 and 143.71)
Human Development Index	
Mean, SD	0.41 (0.14)
Median, Q25 and Q75	0.39 (0.30 and 0.51)

SD: standard deviation, min: minimum, max: maximum, Q25: 25th quintile, Q75: 75th quintile.

## Data Availability

The records of healthcare services in the HIS are publicly available and can be requested through the Ministry of Health’s Public Information Access Portal (https://www.minsa.gob.pe/portada/transparencia/solicitud/frmformulario.asp, accessed on 20 February 2024). Data on the population insured under SIS as of June 2022 can be accessed at https://www.datosabiertos.gob.pe/dataset/datos-de-afiliados-al-seguro-integral-de-salud-en-estado-activo-sis (accessed on 1 April 2024). Lastly, the 2019 district-level HDI can be obtained from the National Center for Strategic Planning (CEPLAN) under the “Datos útiles para el planeamiento” section at https://www.ceplan.gob.pe/informacion-sobre-zonas-y-departamentos-del-peru/ (accessed on 1 April 2024).

## Abbreviations

The following abbreviations are used in this manuscript:

MINSA	Ministerio de Salud
HIS	Health Information System
PSA	Prostate-Specific Antigen
FOBT	Fecal Occult Blood Test
HDI	Human Development Index
CPT	Current Procedural Terminology
RENAES	Registro Nacional de Establecimientos de Salud Acreditados
DIRIS	Dirección de Redes Integradas de Salud
SIS	Seguro Integral de Salud
CEPLAN	Centro Nacional de Planeamiento Estratégico
CC	Concentration Curve
CI	Concentration Index
SD	Standard Deviation
IQR	Interquartile Range
LMICs	Low- and Middle-Income Countries
LAC	Latin America and the Caribbean
DALYs	Disability-Adjusted Life Years
SDG	Sustainable Development Goals
